# Comparison between telelaryngoscopy and suspension laryngoscopy in the diagnosis of benign vocal fold lesions

**DOI:** 10.1016/S1808-8694(15)30147-6

**Published:** 2015-10-18

**Authors:** José Arruda Mendes Neto, Bruno Resende Pinna, José Caporrino Neto, José Eduardo de Sá Pedroso

**Affiliations:** a MD, Otorhinolaryngology Resident - UNIFESP-EPM; b Otorhinolaryngologist. MSc. Student - Laryngology and Voice Department - Federal University of São Paulo Department of Otorhinolaryngology Head and Neck Surgery - Otorhinolaryngologist, MSc student - Larynx and Voice Department - ENT Department - Federal University of São Paulo - UNIFESP; c Otorhinolaryngologist, PhD in Otorhinolaryngology and Head and Neck Surgery - UNIFESP; d Otorhinolaryngologist, PhD in Otorhinolaryngology and Head and Neck Surgery - UNIFESP

**Keywords:** surgery, vocal cords, diagnosis

## Abstract

Proper diagnosis of laryngeal benign lesions still brings doubts among experienced laryngologists, despite current diagnostic progress. **Aim**: the goal of this study was to compare telelaryngoscopy (preoperative) with suspension laryngoscopy (intraoperative) on the diagnosis of vocal fold benign lesions. **Materials and Methods**: We carried out a restrospective study analyzing 79 charts from patients followed up in a University Hospital. In all the charts there was at least diagnostic hypothesis suggested by telelaryngoscopy, which was later on compared to intraoperative findings of suspension laryngoscopy. **Results**: Almost two-thirds of the patients were females, with ages varying between 12 and 66 years (mean of 37 years). Of the 79 patients studied, we diagnosed 95 lesions with telelaryngoscopy and 124 with suspension laryngoscopy. The most frequently found benign lesion was the vocal polyp in both methods. In 64.5% of the cases the diagnosis of the lesions in the outpatient ward was the same as those in the surgical findings. **Conclusion**: Laryngologists must be prepared to alter their surgical planning and treatment approaches because of diagnostic changes that may happen during surgery.

## INTRODUCTION

In 1807, Bozzini was the first author to describe a device used to assess the upper airways. The device was made up of a system of tubes coupled to a mirror with a source of artificial light (candle), used to see the nasopharynx and the hypopharynx.^1^, ^2^

Through the laryngoscope created by Babington, in 1829, one could examine the supraglottic region. In this technique, a small mirror was used to reflect the sun light towards the back of the pharynx, while with the right hand a laryngoscope was positioned in the oropharynx with the goal of visualizing the larynx after pulling the patient”s tongue. However, in his publication, there was no reference about the vocal fold exam, their movement or their function.^1^, ^2^, ^3^

The first description of vocal fold visualization was made in 1855 by Manuel Garcia. In his report, a small mirror attached to a slightly curved rod was placed in the oropharynx, against the soft palate and the uvula of the individual being examined. The larynx was seen through sun light beam reflected on the mirror. In his studies, there are details on vocal fold action during inspiration and vocalization, as well as important observations on how the larynx produced sound.^1^, ^2^, ^3^, ^4^, ^5^

In 1895, Oertel published a paper on the use of the mechanical stroboscope, used to study vocal fold vibration. In 1960 the electronic stroboscopy was described. However, only after the creation of more powerful light sources and with the development of the rigid scopes and micro-cameras, the laryngeal tele-stroboscopy became a truly important tool for the diagnosis of vocal fold lesions.^2^, ^6^

In 1895, Kirstein performed the first direct laryngeal exam by means of a changed aesophagoscope.^2^, ^7^ In 1910, Brunings described the direct microlaryngoscopy with the use of mono-ocular magnification.^2^ In the following year, Killian published the technique for suspension laryngoscopy, allowing the surgeon to have both hands free in order to perform procedures.^2^, ^3^

Tele-laryngo-stroboscopy (TLS) and suspension laryngoscopy (SL) remained as ambulatory exams until the early 20th century. It was only after this period that SL started to be used in the operating room, which contributed much to an improve in the diagnostic accuracy of vocal fold lesions which are not seen by TLS.^2^

Notwithstanding, it was only in 1960 that Scalco performed the first laryngeal microsurgery with the use of suspension laryngoscopy. In the following decade, Kleinsasser divulged this procedure and since then this technique has been considered the treatment of choice for benign lesions of the vocal folds.^2^, ^8^, ^9^, ^10^

TLS remains as an ambulatory exam only, while SL is mostly carried out in the OR, under general anesthesia as a diagnostic and/or therapeutic procedure. They are complementary procedures and have exclusive advantages:

- TLS allows for a real time visualization of glottal closure, besides mucosal wave movement during phonation. It helps assess the vocal ligament with the inspiration maneuver. It is carried out under local anesthesia, and helps select those patients that will benefit from laryngeal micro-surgery.^11^, ^12^
- Through the use of a light microscope, SL gives a superior image of the vocal folds. It allows the physician to palpate the alterations caused in the adjacent soft tissue, thus providing important clues for the diagnosis of glottal lesions not seen through the TLS.^11^, ^12^

Every patient complaining of vocal alteration must be assessed at least by TLS. Dysphonia may be the result of small abnormalities in the mucosal wave vibration of the vocal folds, or secondary to an incomplete glottal closure, or an association of these factors.^12^, ^13^, ^14^.

**Figure 1:**
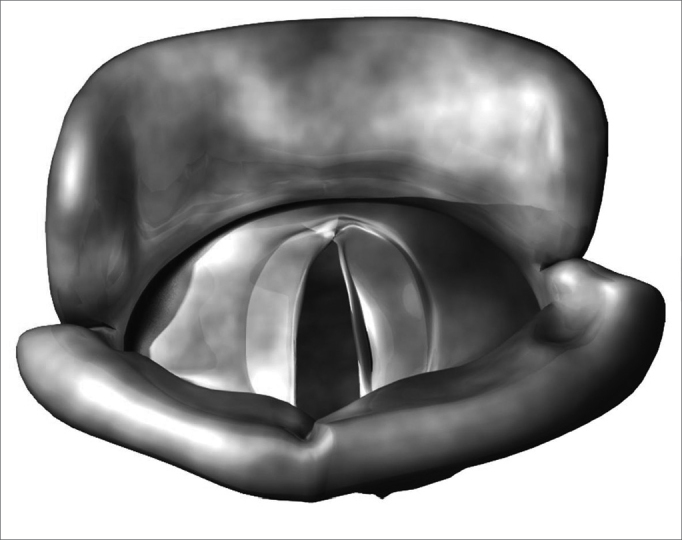
Vocal sulcus.

**Figure 2:**
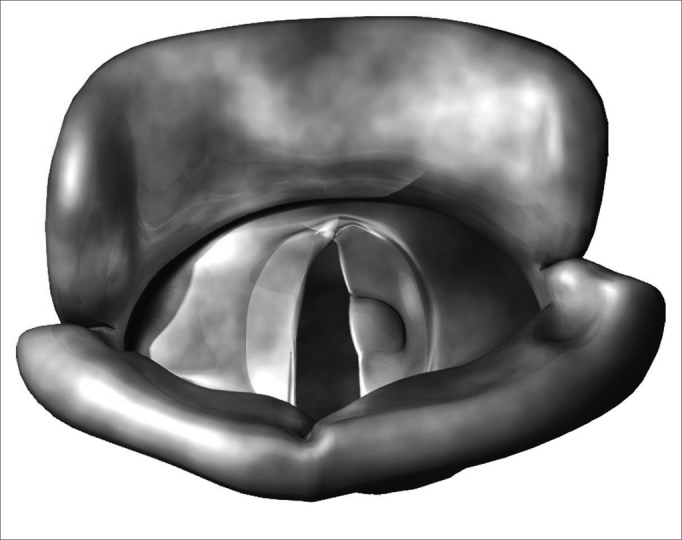
Epidermoid cyst.

**Figure 3:**
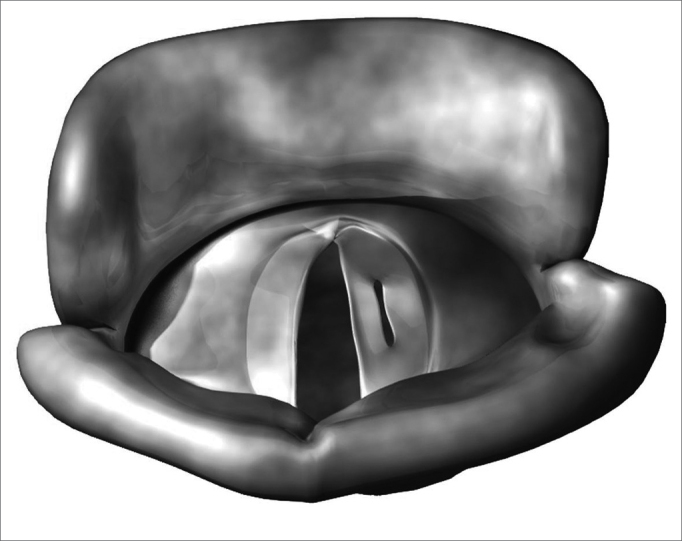
Mucosal bridge.

**Figure 4:**
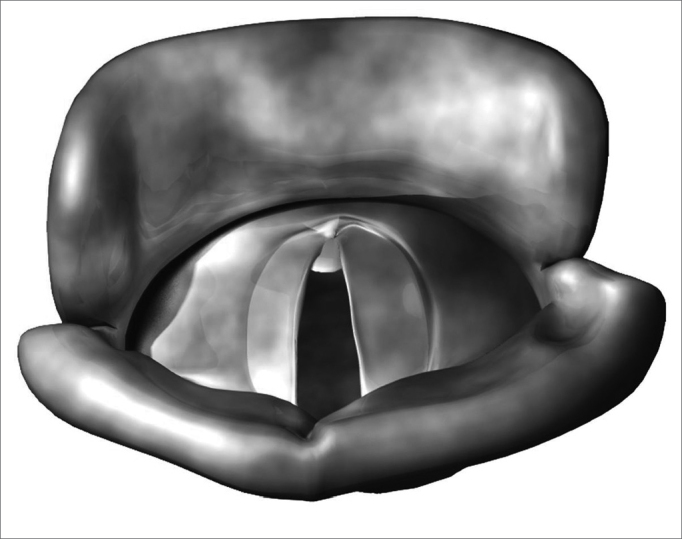
Laryngeal micro diaphragm.

**Figure 5:**
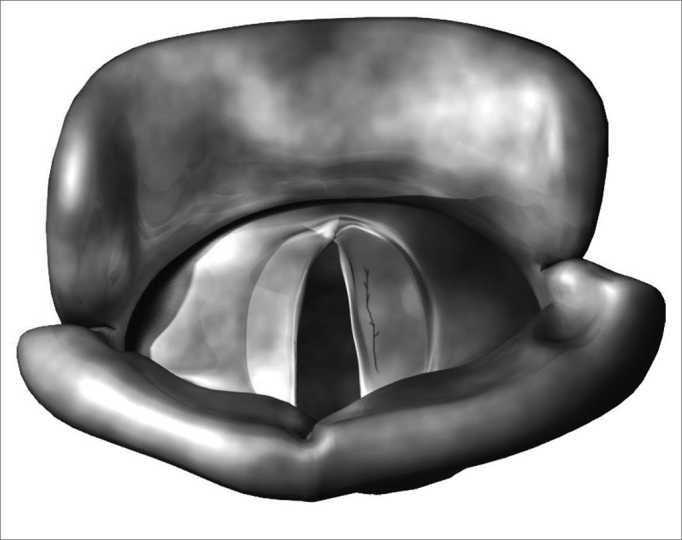
Vascular dysgenesis.

Benign vocal fold diseases can be congenital or acquired because of speech trauma, local irritating agents or neoplasias.^12^, ^13^, ^14^ Minimum structural changes in the vocal fold cover (MSCVF) represent a group of congenital lesions characterized by a tissue disarrangement in the glottal region of which impact is restricted to the laryngeal speech function. According to Pontes, MSCVF can be classified in differentiated and undifferentiated. When undifferentiated, there is no clear macroscopic definition of the lesion during the initial evaluation.^13^ On the other hand, differentiated MSCVF can be classified in five types: (1) vocal sulcus - a vocal fold depression that runs parallel to the vocal fold free border (fig. 1); (2) epidermoid cyst - an epithelial inclusion on the lamina propria (fig. 2); (3) mucosal bridge - alteration initially described by Bouchayer and Cornut15, it is a bundle of lax connective tissue, theoretically identical to the one present in the lamina propria, covered by the same type of stratified epithelial tissue that wraps the vocal fold (Fig. 3); (4) laryngeal micro diaphragm - a small membrane, located on the anterior commisure, inserted on the glottal or subglottic border of the vocal folds (Figs. 4 and 5) vascular dysgenesis - small dilated vessels found on the upper vocal fold surface (Fig. 5).^13^

In some cases, in order to obtain an accurate diagnosis of a benign vocal fold lesion it is necessary to perform suspension laryngoscopy, which also allows for proper dysphonia treatment through microsurgery.^13^

There are very few papers in the literature which compare these two exams. On the other hand, it is common for clinical laryngologists to have initial diagnostic inaccuracy in the assessment of patients with dysphonia.

The goal of the present investigation was:

• Compare the diagnostic reports obtained through TLS and SU;
• Assess the lesions which were not seen during the ambulatory exam;
• Assess TLS diagnostic accuracy.

## PATIENTS AND METHODS

We carried out a retrospective study through the analysis of 79 charts of patients with benign lesions on their vocal folds who were submitted to microlaryngoscopy from January of 2002 to July of 2005, in a university hospital.

The exams (TLS) were carried out by the resident physicians of our department under the supervision of the same three trained professionals in charge of the ward. Pre-operative diagnosis of each patient was carried out through a consensus among these professionals. The materials used for the indirect laryngoscopies were: rigid telescope (8mm) of 70o (MACHIDA, model LY-C30); Bruel & Kjaer stroboscope; Toshiba, model IK-C30A-CCD camera and Sony, model KV-1311CR video monitor. In the TLS assessment protocol, the following was described for each patient: glottal closure; glottal cycles; vocal fold mobility and symmetry; mucosal wave movement and amplitude, and supraglottic activity.

Endolaryngeal microsurgery, with the use of suspension laryngoscopy technique (SL) was also carried out by the resident physicians of our department, under the supervision of two of the ward preceptors. The materials used for surgery were: D.F. Vasconcelos microscope, 400 mm lens, Model IK-C30A-CCD Toshiba camera, Sony, model KV-1311CR video monitor, rigid laryngoscopes with fixators and laryngeal microforceps. During SL, the vocal folds were examined and, besides visual inspection, the region was also palpated with delicate microforceps. One cordotomy was carried out following the diagnosis of a benign lesion to be treated surgically. Now, in the cases of lesions without surgical indication the patients were referred to speech therapy.

Cover minimum structural alterations and speech trauma lesions were diagnosed through these two methods. Of the undetermined MSCFV, one alteration described as “contralateral reaction” was separately assessed in this group, since it deals with a lesion secondary to repeated trauma of a deformity on the other vocal fold. In the charts studied, we did not see a description of vascular dysgenesis or laryngeal micro-diaphragm. Among the speech trauma lesions we stress vocal nodules, Reinke”s edema and vocal polyps. Vocal nodules were not included in the initial diagnostics made by TLS, because during the period studied, all evolved satisfactorily with speech therapy, and surgical intervention was unnecessary. However, some lesions, which previously presented other diagnoses, were classified as vocal nodule after SL. There was no diagnostic doubt in relation to Reinke”s edema cases. In all cases of such disorder analyzed, there was no difference between indirect laryngoscopy and the microsurgery finding, thus, these patients were taken off the series.

In this study, the diagnoses varied from one pre-op lesion to one or more intraoperative lesion or no lesion at all with TLS for one (or more) with SL.

The assessment of accuracy for each telelaryngoscopy exam was done through the following criteria: accurate - if the diagnosis of the lesion identified in the pre-op with TLS were confirmed with SL and, inaccurate - if the alterations seen at TLS were not identified by SL, or if a lesion were identified only in the intraoperative.

Ethics committee - protocol #2007110521105

## RESULTS

**Chart 1:**
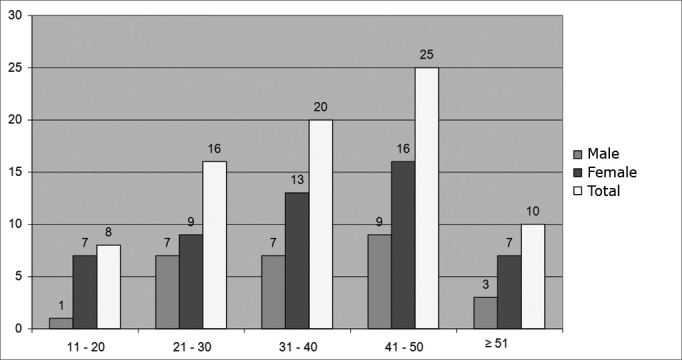
Distribution in absolute numbers of gender by age range in years (n=79 patients).

We assessed 79 patients, 52 women (65.8%) and 27 men (34.2%). Mean age was 37 years, varying from 12 to 66 years, according to Chart 1.

**Chart 2:**
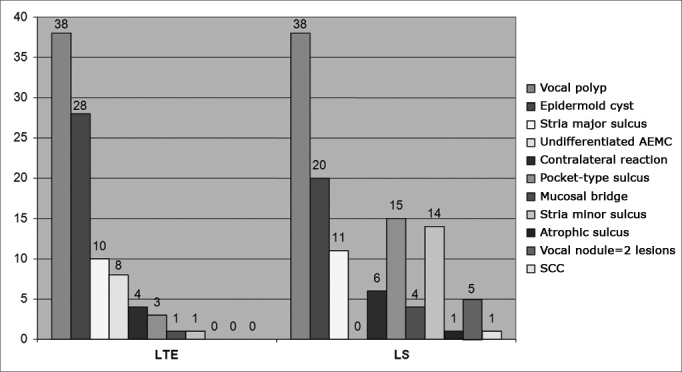
Distribution in absolute number of diagnosis before (n=95) and after SL (n=124).

We diagnosed 95 lesions with telelaryngoscopy in the patients studied. In 16 of them (20.2%), there were alterations in the two vocal folds (32 lesions), while in 63 patients (79.8%), we observed lesions in only one of the vocal folds. With this test, the most frequent diagnosis was of vocal polyp (40%), followed by epidermoid cyst (29.4%) and stria major sulcus (10.5%). MSCFV were the least frequently found alterations: stria minor sulcus - 1% and mucosal bridge - 1% (Chart 2).

We observed 124 lesions through suspension laryngoscopy. Of the 79 patients, 48 (60.7%) had only one lesion on their vocal folds. On the rest of them - 39.3%, there were at least two alterations in their glottal regions. Two patients had four lesions in their vocal folds. We diagnosed vocal polyps (30.6%), epidermoid cyst (16.1%), pocket-type sulcus (12.1%), and stria minor sulcus (11.3%), amongst other. All cases classified as undifferentiated MSCFV in the pre-op changed diagnosis with SL (Fig. 2).

Besides the 95 initial lesions, we observed 29 more alterations with the SL (23.3%). The most frequent diagnoses in this case were: stria minor sulcus (37.8%), pocket-type sulcus (17.2%) and stria major sulcus (10.3%).

TLS accuracy was observed in 100% of the cases of mucosal bridge, stria minor sulcus and sulcus pocket. In vocal polyps (total of 38 lesions), the rate of correct diagnosis was of 73.6%, in other words, 10 lesions (26.4%), which were initially described as vocal polyp with TLS and later changed diagnosis with the SL. Of the 28 lesions identified as epidermoid cyst in the physical examination, 14 (50%) were truly vocal cysts. Of these 14, nine were pocket-type sulcus (Tables 1 and 2). Tele-laryngo-stroboscopy”s global accuracy was of 64.5% (51 correct in 79 patients).

**Table 1:** TLS diagnostic accuracy for each benign lesion of the vocal folds.

Lesion	Diagnostic method		TLS diagnostic accuracy
	Preoperative TLS	Intraoperative SL	
Pocket-type sulcus		3	100%
Stria minor sulcus	1	1	100%
Mucosal bridge	1	1	100%
Polyp	38	28	73,6%
Stria major sulcus	10	6	60,0%
Cyst	28	14	50,0%
Granuloma	2	1	50,0%
Contralateral reaction	4	2	50,0%
Undifferentiated MSCFV	8	0	0%

**Table 2:** Inaccuracy in outpatient diagnosis.

Diagnosis through LS											
Diagnosis through LTE	N^o^	Vocal polyp	Eppidermoid cyst	Granuloma Reação contra lateral	Sulco estria Major	Minor	Mucosal bridge	Vocal nodule	Pocket-type sulcus	SCC	Others*
Epidermoid cyst	14					1		2	9		1
Vocal polyp	10		2	1				1	1	1	4
Undifferentiated AEMC	8	2			1	1	2	2			
Stria major sulcus	4			1					2		1
Granuloma	1							1			
Contralateral reaction	2		1								1

No – Number of lesions with innacurate diagnosis through TLE.

* Lesion not seen through SL or incorrect lesion diagnosis on the contralateral vocal fold

Of the eight undifferentiated MSCFV seen during physical exam, we later diagnosed, through SL, two mucosal bridge lesions, two vocal polyps, one stria minor sulcus, one stria major sulcus and one case of vocal nodule, described on Table 2.

The atrophic sulcus diagnosis was carried out during surgery. Moreover, in five cases, after SL, the alterations initially described as cyst (unilateral in two cases), polyp (unilateral in one case), granuloma (unilateral in one case) and undifferentiated MSCFV (bilateral in one case) were reclassified as vocal nodules. In ambulatory follow up of one patient after surgery, the lesion initially described as vocal polyp, housed an in situ squamous cell carcinoma (SCC) according to the histopathology result (Table 2).

## DISCUSSION

TLS accurate diagnosis was the main objective of this study. Ambulatory diagnoses were made up based on tele-laryngo-stroboscopy. By observing and palpating the lesions during surgery, we obtained the diagnosis of vocal fold alterations with SL.

We observed a predominance of female patients in this study (65.8%). Such rate is expected because of the greater concern women have in relation to mild voice changes. Dailey et al., in 2007, a larger number of females complaining of dysphonia was also seen (62%).^11^ Similar data was found by Poels et al., in 2003.^12^ In 1999, Pontes et al. published that women represented 65% of the patients with MSCFV of the vocal folds.^16^

Analyzing gender distribution according to age range, fewer males were seen in among all ages studied. Nonetheless, Poels et al., in 2003, published a higher prevalence of men among patients above 30 years of age.^12^

We observed unilateral lesions in 79.8% of the patients using TLS; and 60.7% with SL. Poels et al., in 2003, published rates of 43% and 18%, respectively.^12^ This difference may have been attributed to the initial exclusion of bilateral disease, such as vocal nodules and Reinke”s edemas in the present study.

The most frequently found lesions in the outpatient ward were vocal polyps (40%) and epidermoid cyst (29.4%). Excluding vocal nodules, the results were similar to the ones found by Dailey et al., in 2007, Poels et al., in 2003 and Colton et al., in 1995.^11^, ^12^, ^17^

During microsurgery, 124 lesions were described in the 79 patients. The most frequent diagnoses with SL were vocal polyps (30.6%), epidermoid cysts (16.1%), pocket-type sulcus (12.1%) and stria minor sulcus (11.3%). We observed 29 more alterations (23.3%), not seen in the preoperative, especially stria minor sulcus. These results were similar to the ones in the literature, when we exclude vocal nodules and Reinke”s edema.^11^, ^12^

We observed a great TLS accuracy (100%) in the diagnosis of stria minor sulcus, pocket-type sulcus and mucosal bridge. The rate of correct diagnoses for stria minor sulcus was of 78.4% in the study carried out by Poel et al., in 2003 (no cases of mucosal bridge or pocket-type sulcus were described in this author”s paper). In these cases there is a great likelihood that these MSCFV could be seen on SL, especially the cases of stria minor sulcus.^12^

Diagnostic precision regarding vocal polyps and epidermoid cysts were of 73.6% and 50%, respectively. The vocal polyp was initially interpreted as cyst in two patients, while in four other cases the TLS was not considered accurate, because the contralateral lesion to the polyp seen was not properly diagnosed by the SL. Of the 28 cysts, nine were pocket-type sulcus. In 2003, Poels et al., described rates of 70% and 20%, respectively.^12^ In this paper, polyps were mistaken with cysts and vocal nodules, while the epidermoid cyst was mistaken by vocal sulcus and vocal polyp. Besides SL, a good means of making the correct differential diagnosis between polyps and cysts is through the correct use of videostroboscopy and its proper functioning. The use of this test, presented by Shohet et al., in 1996, showed a reduction of the mucosal wave in 100% of the patients with epidermoid cyst, while in the case of polyps, the mucosal wave was present, or even increased in up to 80% of the cases studied.^18^

There was a change in the initial diagnosis in 35.5% of the cases. The major reasons behind these inconsistencies were the lesions initially described as epidermoid cyst and undifferentiated MSCFV, reclassified with the SL as mucosal bridge, vocal polyp, stria minor sulcus, stria major sulcus and vocal nodule. In 2003, Poels et al. noticed that in 36% of the cases followed up there were changes in the intraoperative diagnoses. The main interpretation errors were between vocal nodules and vocal sulcus, as well as with epidermoid cyst and vocal sulcus.^13^

Some explanations can be given to explain these diagnostic changes:

- In 2003, Poels et al. and Pontes et al., in 1999, observed that the frequent associations between vocal polyp and the contralateral reaction; cyst and the contralateral reaction; leukoplasia and MSCFV; vocal fold inflammation and MSCFV make it difficult to properly visualize these lesions during outpatient exam.^13^, ^16^
- According to Monday et al., in 1983, the unilateral inflammation of a vocal fold has contributed to the inaccurate diagnoses of cysts.^19^
- The correct diagnosis of a lesion with TLS depends on the proper calibration of the stroboscope.^13^
- The long line of patients waiting for surgery in a university hospital keeps the outpatient ward consultation and the time of surgery very far apart, and this may contribute to a change in the previous lesion or the appearance of a new lesion because of inadequate voice use.
- Variation in the interpretation of the outpatient exam and the SL among different observers or among different observation moments by the same professional. ^13^
- The very diversity of nomenclature found in the literature.^11^

In five patients, two lesions previously described as epidermoid cyst and others as undifferentiated MSCFV, granuloma and polyp were reclassified as vocal nodule. When there is edema in the lower lip of a bilateral stria minor, TLS can not differentiate it from the vocal nodule. In 2003, Poels et al., described results similar to the ones found in this study.^13^

In one case only, a vocal polyp housed a SCC. Every lesion was resected and referred to the Department of Pathology for a histological analysis.

## CONCLUSION

The main diagnoses found in this study were polyps and cysts, regardless of the method used. The epidermoid cyst was the lesion which, in absolute numbers, presented the greatest diagnostic variation when seen by SL.

The main lesions undiagnosed during the outpatient exam were stria minor sulcus, pocket-type sulcus and stria major sulcus.

The accuracy of the outpatient diagnosis of vocal fold lesions was observed in 64.5% of the patients submitted to TLS.

Because of these differences between the preoperative and intraoperative findings, the laryngologist must be prepared to change surgical planning and the therapeutic approaches when needed. Moreover, he/she must clearly educate the patient about the possibility of change in the previously established approach, since the alterations seen during suspension laryngoscopy can differentiate those seen in the office.
